# Seroprevalence of *Toxoplasma gondii* infection in feral cats in Qatar

**DOI:** 10.1186/s12917-017-0952-4

**Published:** 2017-01-18

**Authors:** Sonia Boughattas, Jerzy Behnke, Aarti Sharma, Marawan Abu-Madi

**Affiliations:** 1 Department of Biomedical Science, College of Health Sciences, Biomedical Research Center, Qatar University, P.O. Box 2713, Doha, Qatar; 2 School of Biology, University of Nottingham, University Park, Nottingham, NG7 2RD UK

**Keywords:** Serosurvey, Antibodies, Toxoplasmosis, Felids, Middle East, Season, Sex

## Abstract

**Background:**

Cats are essential in the life cycle of *Toxoplasma gondii* as they can shed the environmentally resistant oocysts after acquiring infection. Human populations living in cities with high densities of feral cats are therefore likely to be at risk of infection. The current study is the first to estimate the seroprevalence of *T. gondii* in the feral cat population in Qatar. We investigated the seroprevalence of *T. gondii* among 495 adult cats from urban and suburban districts in Qatar. Using results from the Modified Agglutination Test, we fitted statistical models with host sex, area and season as explanatory factors and seropositivity as the outcome.

**Results:**

The analysis revealed an overall seroprevalence of 82%. Seroprevalence was significantly higher in the summer season (*P* = 0.006). No significant difference was detected (*P* > 0.05) between seroprevalence in female and male cats and in cats from urban and suburban districts of Qatar.

**Conclusions:**

Despite the seasonal difference, the observed seroprevalence of *T. gondii* suggests high environmental contamination throughout the year, with some female cats generating more intense responses compared to males. Both findings merit further investigations.

## Background

Toxoplasmosis is a widespread zoonotic parasitic infection. It is estimated that about one third of the world population is chronically infected with *Toxoplasma gondii*.

Felids are the only definitive hosts of the parasite and can shed millions of environmentally resistant oocysts in their feces if they become infected [1]. The oocysts can remain infectious for more than 1 year in unfrozen and moist soil [2] and can cause outbreaks of toxoplasmosis. This is evidenced by the large waterborne outbreak of toxoplasmosis in Canada in 1995 that was epidemiologically linked to oocyst contamination of a water reservoir in British Columbia [3].

Cats were introduced to Qatar in the 1960s to control the high rodent population in the country, but subsequently they in turn reproduced rapidly [4]. The current density of cats in Qatar may pose a risk for humans, as cats are natural hosts for a wide range of zoonotic pathogens, including *T. gondii*. The overall seroprevalence of *T. gondii* among the Qatari human population has been estimated at 29.8% with a progressive rise from <4% in the 1 year old group to 41.2% at >45 years of age [5]. Such observations provide further evidence for the increased risk of infection with increasing age through longer exposure time.

As cats shed *Toxoplasma* oocysts for only a short period of time after initial infection, detection of patent infections in cat populations (based on faecal oocyst counts) is likely to underestimate the number of animals that have been exposed to infection [1]. However, *T. gondii* elicits strong antibody responses in its hosts and therefore assessment of seroprevalence is an alternative approach for studying the epidemiology of this pathogen. Estimates of antibody prevalence in the cat population also provide another useful indicator of environmental contamination [6]. The current study is the first to estimate the seroprevalence of *T. gondii* in the feral cat population in Qatar.

## Methods

### Sample collection

Feral cats were caught live as part of the routine activities of the Qatar Cat Control Unit as described previously [4]. Briefly, trapped adult cats that were eligible for the trap-neuter-return program were transported to a shelter for sterilization. For each animal the sex, the area, and the season of sampling were recorded. In the current study, sampling began in September 2014, and ended in September 2015 (394 samples in summer and 101 samples in winter).

Cats from both urban (*n* = 216) and sub-urban (*n* = 279) areas were investigated in this study. Within each sector, traps were set out in places most likely to be frequented by cats at night, i.e. in alleyways, in back yards close to houses, and near to rubbish bins and municipal garbage containers.

Blood was drawn by experienced veterinarians complying strictly with animal welfare guidelines as stipulated by the National Institutional Animal Care and Use Committee of Qatar. Blood samples were then centrifuged at 5000 × *g* for 20 min at room temperature and the resulting supernatants were collected and used for serology.

### Serology

A total number of 495 sera (234 males and 261 females) were tested for *T. gondii* IgG antibodies using commercially available *Toxoplasma gondii* antigen (#EH2001, Kerafast® Boston, USA). Two-fold serial dilutions, in phosphate buffered saline (pH 7.2), were made from 1:25 to 1:200 and tested with a Modified Agglutination Test (MAT), as previously described [7]. Samples that were positive at a dilution of 1:200 were further diluted in a second run to a dilution of 1:3,200 for antibody titration, and those positive at 1:3,200 were diluted in a third run until no further agglutination was evident.

Positive controls (provided by the Department of Laboratory Medicine and Pathology at Hamad General Hospital) and negative controls (serum dilution buffer without serum) were included in each test. Agglutination in at least half of the “U” well bottoms of a microplate was accepted as a positive reaction. In the case of complete sedimentation on the well bottom with no sign of agglutination, the sample was recorded as negative, i.e. no evidence of *T. gondii* antibodies in the serum. A titer (inverse of a dilution) of 25 or higher was considered positive.

### Statistical analysis

Prevalence values (percentage of animals infected) are given with 95% confidence limits (CL_95_), calculated by bespoke software based on the tables of Rohlf and Sokal [8]. For analysis of seroprevalence, we used maximum likelihood techniques based on log linear analysis of contingency tables in the software package IBM SPSS Statistics Version 21 (IBM Corporation). Initially, full factorial models were fitted, incorporating as factors SEX (at 2 levels, males and females), AREA (at 2 levels, 1 for urban areas [Al Hilal, Bin Omran, Al Sadd, Umm-Ghwailina, Al Salata, Madinat Khalifa, Al Nasr, Al Maamoura, Al Dafna, Al Muntzah, Al Markhiya, Al Najma, Old Airport, Al Asiri] and 2 for suburban areas [Al Azizia, Al Rayyan, Al Luqta, Al Waab, Abu Hamour, Al Wakrah, Muaither, Um Al Amad, Shahaniya, Al Gharrafa, Al Kharaitiyat, Umm Salal, Al Zaghwa, Al Wajba, Al Khor, Al Khissa]) and SEASON (at 2 levels, summer [May-October] and winter [November-April]). INFECTION, reflecting the presence or absence of antibodies to *T. gondii* (overall or at specific dilutions) was coded as a binary factor, and the cut-off for statistical significance was considered to be a *P* value of 0.05 (two tailed) [4].

For analysis of quantitative data we first ranked the titers on a scale from 0 to 8, where 0 = no agglutination, 1 = maximum agglutination at 1:25 and so on to 8 = agglutination at dilutions higher than 1:3,200. We used a zero-inflated model in R version 2.2.1 (R Core Development Team and the *pscl* package) that analyzed a binomial process (0, 1 for the high responders versus the rest with a binomial model with logit link) and a Poisson (Poisson model with log link) for the rest.

## Results

**Fig. 1:**
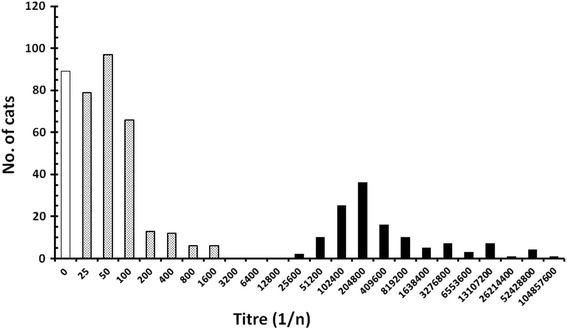
Frequency distribution of antibody titres among the sampled cats. The figure shows the number of cats for which no antibody was detected (first column filled in white) and those that had detectable antibody. Titres for the latter are given as the reciprocal of the maximum dilution (n) at which agglutination was detected and these fall into two peaks. Columns filled with stippled shading indicate the numbers of cats with titres in the lower intensity antibody range and those filled in black represent cats with the higher titres

Of the 495 feral cats (234 males and 261 females) tested, 406 (82.0%, CL_95=_76.11–86.79) were positive for *T. gondii* antibodies. Four samples presented prozone effects with negative results at the low dilution of 1:25 and positive agglutination at higher dilutions ≥ 1:1,600. The frequency distribution of antibody titers among the sampled cats is shown in Fig. 1, and the bimodal distribution of antibody titres, with peaks in the lower and top ends of the positive range can be clearly seen.

**Fig. 2:**
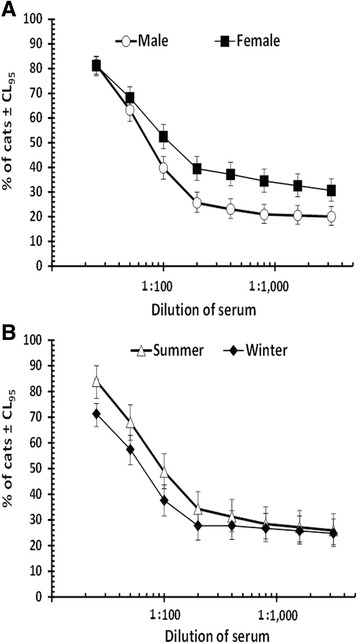
The effect of host sex and season on the percentage of cat sera showing agglutination at varying dilutions of serum. Percentage values (percentage of animals with evidence of agglutination) are given with 95% confidence limits (CL_95_) for each level of serum dilution. **a** Effect of host sex (for male cats *n* = 234, and for females 261 at each serum dilution). **b** Effect of season (in the summer *n* = 394 and in the winter 101 at each serum dilution). For statistical analysis see text

Seroprevalence of *T. gondii* was similar in males and females: 82.5% (CL_95_ = 78.58–85.84) and 81.6% (CL_95_ = 77.45–85.17) respectively. With SEASON and AREA taken into account, the difference between the sexes was not significant (for SEX x INFECTION *χ* ^2^_1_ = 0.216, *P* > 0.05). However, when the prevalence of antibodies in male and female cats was plotted separately against serum dilution (Fig. 2a), there was a significant effect of host SEX at high antibody titers (models taking SEASON and AREA taken into consideration, at a serum dilution of 1:400, for SEX x INFECTION *χ* ^2^_1_ = 11.69, *P* = 0.001; and in serum dilutions higher than 1:3,200; *χ* ^2^_1_ = 7.30, *P* = 0.007).

Seroprevalence was similar in cats from urban and sub-urban areas (82.4%, CL_95_ = 78.64–85.67, and 81.7%, CL_95_ = 77.42–85.37, respectively) and no significant difference between areas was detected whether testing overall positivity (for AREA x INFECTION *χ* ^2^_1_ = 0.42, *P* > 0.05), or positivity at specific dilutions of serum.

The number of cats sampled in the summer was 394, and 101 in the winter months. There was a significant effect of SEASON on seropositivity (with host SEX and AREA taken into account, for SEASON x INFECTION *χ* ^2^_1_ = 7.52, *P* = 0.006). Seroprevalence was higher in the summer months (84.5%, CL_95_ = 79.62–88.46) in contrast to the winter (72.3%, CL_95_ = 65.53–78.14 and see Fig. 2b).

Quantitative analysis was in general in agreement with these conclusions. Based on the zero-inflated model and a binomial process in which the high responders (cats showing titers in excess of 1:3,200) were coded as 1, and the rest as zero, the occurrence of the highest titers (>1:3,200) was affected significantly only by host sex (*z* = 2.534, *P* = 0.0113). Males (mean rank = 0.201 ± 0.025, *n* = 234) had a lower frequency of high titers than females (mean rank =0.307 ± 0.029, *n* = 251), as found in the earlier log linear model given above.

## Discussion

This study demonstrated a high overall seroprevalence of *T. gondii* antibodies in the feral cat population in Qatar. Seroprevalence was higher than values reported from neighboring countries, as for example 19.6% in Kuwait [9] and 30.4% in Iraq [10]. Similar seroprevalences to those recorded in this study have been observed mainly among cats from tropical areas: Ethiopia with 85.4% [11] and the Amazon with 87.3% [12].

While the present work did not find an overall significant difference in seroprevalence between male and female cats as observed elsewhere [13, 14], higher antibody titers were detected in female cats when analysis was restricted to the high dilutions (>1/400) which is in accordance with other reports [6, 15]. However our observation of some female cats expressing more intense antibody responses than male cats contrasts with the conclusions reached by Miro et al. [16], who reported in their study that stray male cats had significantly higher seroprevalence. The authors linked their findings to differences in the territorial habits of male and female cats, as male cats are more likely to wander and thus experience more access to contaminated sources [17]. Although ecological, social and epidemiological factors may account for some of the female biased antibody responses in our study, hormone-influenced immunological mechanisms have been proposed as the more likely mechanism underlying sex-biased parasitism with *T. gondii* [18].

Cat bioassays have not provided any clear evidence of sex-bias in the occurrence of toxoplasmosis [19]. However, experimental studies in mice have shown that females are more susceptible than males to *T. gondii* infection and develop more severe cerebral inflammation. They are more likely to die following infection than males [20]. Female mice that survived the acute phase and developed chronic infections harbored more cysts in their brains than did surviving males. Gonadectomy of female mice was shown to reduce the development of tissue cysts caused by *T. gondii* infection, whereas estrogen administration was found to exacerbate the infection [21].

It remains unknown when the cats that tested positive had acquired the infection. The low intensity responses (≤1/400) which were recorded in more than half of the cats in our study suggest antibodies from a past infection but may also include cats that have very recently been exposed to the parasite, and have not yet developed the maximum response. The cats that showed intense antibody responses (≥1/800 dilution) are more likely to be cats that had acquired the infection recently but after a sufficient time to mount the response [22]. Since the prevalence of high intensity antibody responses was almost identical in both winter and summer months, this indicates that there is a persistent source of *T. gondii* infection in the environment in which the cats live. Moreover, the consistently high seroprevalence in both urban and sub-urban areas of Qatar suggests a high level of *T. gondii* contamination throughout the country.

The large home ranges of feral cats, which can stretch to 10 km^2^, implies widespread contamination of the environment, especially given that some reports have documented cats travelling up to 45 km in just 2 days [23]. Domestic and stray/feral cats are the same species (*Felis catus*) and the main difference between them is their lifestyle. In contrast to domestic animals, feral cats are unowned and live in the streets, alleys, farm buildings, factories, wharves or abandoned vehicles. They may be semi dependent on humans from whom they may receive some food. It has been reported elsewhere that 76.5% of cats fed table leftovers are *Toxoplasma* seropositive with a majority showing high IgG antibodies titers [24]. These free-roaming cats are exposed to a wide variety of pathogens, and have been shown to be excellent sentinels of infectious and parasitic diseases. Consequently they provide useful information on circulation of pathogens in domestic and wild ecosystems [13]. Most cats are thought to become infected with *T. gondii* after weaning when they begin to hunt for food. Outdoor access facilitates hunting behavior, as in the Netherlands where 93% of the cats with free outdoor access exhibited hunting behavior [22]. Stray cats and cats with outdoor access usually acquire the infection from hunting rather than from the ingestion of oocysts [25]. Hence the recommendation to pet owners that to protect their cats from *T. gondii* infection the hunting of small prey should be discouraged/prevented, and that all meat served to their pet cats should be thoroughly cooked and/or frozen before cooking [26].

In a study conducted in Italy, it was suggested that infected intermediate hosts that are prey for cats are more available in the summer season [27]. A recent analysis reported a correlation between the geolatitude (which strongly correlates with temperature and quantity and quality of sunlight) and humidity (which favors the survival of *Toxoplasma* oocysts in soil) and occurrence of toxoplasmosis [28]. The efficiency of transmission may therefore differ between seasons in Qatar, with a higher risk of exposure in the summer months (where the climate is more suitable for survival of the oocysts). Qatar typically experiences two seasons. In the summer season, daytime temperatures frequently exceed 45 °C and seldom fall below 18 °C at night. Humidity peaks in August at 90%, before subsiding to 70% towards the end of October [4]. The latter months of the summer season certainly fall within this definition of the optimal climatic conditions for transmission. During the winter season temperatures peak at about 28 °C and may fall as low as 7 °C at night and the mean humidity is usually in the range 75–86%. These conditions are less suitable for survival of oocysts of *T. gondii* in the external environment. Thus, the tenacity and infectivity of oocysts in winter may be less than in summer, and hence transmission to cats and potential intermediate hosts such as rodents may be less efficient in winter months. However, this requires further studies, for instance by environmental sampling for *T. gondii* oocysts and thorough assessment of the resilience of oocysts of local isolates of *T. gondii* to typical summer and winter conditions in Qatar. Unfortunately, most people are unaware that they can acquire toxoplasmosis from the environment by direct contact with soil or water [29].

## Conclusions

The seroprevalence of *T.gondii* in cats in Qatar is high. Our data showed that female cats were more likely to have high antibody responses compared with males, and that seroprevalence is higher in the summer months. Overall, our data imply high contamination of the local environment in Qatar and we recommend further investigation of food/water sources through which transmission may occur.
